# Working Memory after Traumatic Brain Injury: The Neural Basis of Improved Performance with Methylphenidate

**DOI:** 10.3389/fnbeh.2017.00058

**Published:** 2017-04-05

**Authors:** Anne E. Manktelow, David K. Menon, Barbara J. Sahakian, Emmanuel A. Stamatakis

**Affiliations:** ^1^Division of Anaesthesia, University of CambridgeCambridge, UK; ^2^Wolfson Brain Imaging Centre, Department of Clinical Neurosciences, University of CambridgeCambridge, UK; ^3^Department of Psychiatry, MRC/Wellcome Trust Behavioural and Clinical Neuroscience Institute, University of CambridgeCambridge, UK

**Keywords:** traumatic brain injury, working memory, methylphenidate, functional connectivity, cerebellum, fMRI, cognitive function

## Abstract

Traumatic brain injury (TBI) often results in cognitive impairments for patients. The aim of this proof of concept study was to establish the nature of abnormalities, in terms of activity and connectivity, in the working memory network of TBI patients and how these relate to compromised behavioral outcomes. Further, this study examined the neural correlates of working memory improvement following the administration of methylphenidate. We report behavioral, functional and structural MRI data from a group of 15 Healthy Controls (HC) and a group of 15 TBI patients, acquired during the execution of the N-back task. The patients were studied on two occasions after the administration of either placebo or 30 mg of methylphenidate. Between group tests revealed a significant difference in performance when HCs were compared to TBI patients on placebo [*F*_(1, 28)_ = 4.426, *p* < 0.05, η_*p*_^2^ = 0.136]. This difference disappeared when the patients took methylphenidate [*F*_(1, 28)_ = 3.665, *p* = 0.66]. Patients in the middle range of baseline performance demonstrated the most benefit from methylphenidate. Changes in the TBI patient activation levels in the Left Cerebellum significantly and positively correlated with changes in performance (*r* = 0.509, *df* = 13, *p* = 0.05). Whole-brain connectivity analysis using the Left Cerebellum as a seed revealed widespread negative interactions between the Left Cerebellum and parietal and frontal cortices as well as subcortical areas. Neither the TBI group on methylphenidate nor the HC group demonstrated any significant negative interactions. Our findings indicate that (a) TBI significantly reduces the levels of activation and connectivity strength between key areas of the working memory network and (b) Methylphenidate improves the cognitive outcomes on a working memory task. Therefore, we conclude that methylphenidate may render the working memory network in a TBI group more consistent with that of an intact working memory network.

## Introduction

Traumatic brain injury (TBI) is heterogeneous in nature. TBI patients have enormous variation in the location and severity of injury, resulting in a wide range of outcomes. Even “good” outcomes may result in significant levels of cognitive impairment which affect the patient's quality of life.

TBI can involve lesions to cortical areas or more diffuse damage involving white matter tracts that connect cortical areas. This type of white matter injury [diffuse axonal injury (DAI)] is common in the majority of trauma to the head as the rotational and shearing effects of road traffic accidents and falls cause extensive damage to the axonal (white matter) pathways that connect different parts of the brain (Povlishock and Katz, 2005; Johnson et al., 2013). TBI predominately affects the frontal lobes, regardless of the mechanism of injury and subsequent pathophysiology (Stuss, 2011), and this may result in deficits in a range of cognitively demanding tasks including executive control, working memory, episodic memory and problem solving as well as processing speed (Levin et al., 1990; Mazaux et al., 1997; Salmond and Sahakian, 2005; Parente et al., 2011).

In this study we used functional MRI (fMRI) versions of several tasks to interrogate different aspects of cognition that could be affected by TBI including the Stop Signal task (response inhibition), Rapid Visual Information Processing task (sustained attention) and the Tower of London task (planning). In this paper we will focus on the analysis of the N-back task to investigate working memory (WM) in TBI patients by comparing them to a group of healthy controls (HC).

WM is a processing system that allows for the maintenance and manipulation of information for temporary use without the information being encoded into short or long term memory storage. The main theoretical account of WM processing was developed by Baddeley (1986, 2007) who suggested three main components, including the central executive, which is responsible for manipulation of information; and two storage (maintenance) systems, which are specific to language (the phonological loop) and spatial information (the visuospatial sketchpad) (Klauer and Zhao, 2004; Baddeley, 2012).

This theoretical model has been extensively studied and the advancement of functional brain imaging techniques has allowed for the investigation of its neurobiological correlates. In healthy volunteers, WM tasks activate bilateral frontal areas, bilateral parietal areas and parts of the superior temporal lobe (Cohen et al., 1997; Owen, 1997; Owen et al., 1999; Fletcher and Henson, 2001). The WM network also includes brain areas associated with language for verbal WM tasks (Broca's area and inferior supramarginal gyrus) (Paulesu et al., 1993) and areas associated with vision with spatial/visual WM tasks (superior occipital gyrus, right calcarine, bilateral fusiform gyrus) (Salmon et al., 1996). There is now increasing evidence that the cerebellum plays an integral part in the WM network. Desmond et al. (Desmond et al., 1997; Chen and Desmond, 2005a,b) demonstrated that bilateral areas in the superior hemispheres of the cerebellum are activated in a load-dependent manner during a verbal rehearsal task. In contrast, the right inferior cerebellar hemisphere was activated only during the WM maintenance stage of the same task, along with concurrent increases in activation in the left inferior parietal lobe.

Investigations of compromised WM networks can help to refine our thinking on how this network is organized in the healthy brain. Previous studies of brain activation and function post TBI indicate that patients recruit right hemisphere regions (particularly in the frontal lobe) during WM task execution, in contrast to HCs who primarily activate left frontal regions during WM tasks (Christodoulou et al., 2001; Perlstein et al., 2004; Kasahara et al., 2011). A recent theory, based on the alterations in cerebral activation seen in TBI patients, postulates that the recruitment of cognitive resources from the contralateral hemisphere is greater and occurs earlier during a cognitive task than in the HCs (Hillary et al., 2011). These same areas in the right frontal and parietal cortex would also be utilized by HC while carrying out a WM task but mostly at much higher cognitive loads (Hillary et al., 2011; Medaglia et al., 2012). There is also evidence that, with time and practice, the activation patterns for TBI patients during a WM task can more closely approximate the activation patterns of the HCs (Sanchez-Carrion et al., 2008). Taken together, these results suggest that TBI patients may be able to recover some of the normal WM network function and activation patterns by tapping into “latent” resources over time and with task practice.

The main research question we aimed to answer with our study was whether WM performance deficits in TBI patients reflected distinct neural changes as seen in activation profiles and functional connectivity of the WM network. Furthermore, we explored changes in the patient activity profile and network integration following the administration of the cognitively enhancing drug Methylphenidate (MPh).

Beneficial effects on cognitive performance have been observed with the administration of MPh in HCs for tasks that examined spatial WM, planning (Elliott et al., 1997) and WM (Mehta et al., 2000) where volunteers with a lower baseline WM capacity demonstrated greater performance improvement. However, MPh has also been shown to have differential effects on WM task performance in HCs with multiple cognitive demands. Fallon et al. (2017) used a delay match-to-sample WM task which involves both distraction and updating to examine cognitive stability and cognitive flexibility, respectively. They found that MPh improves cognitive stability associated with BOLD signal changes in the prefrontal cortex (PFC) but MPh impaired the updating of information held in WM (cognitive flexibility). The conclusion drawn by Fallon et al. (2017) was that MPh works by modulating dopamine enhancement in the striatum which, in turn, influences cognitive function in the PFC resulting in the improvement of one cognitive function at the expense of another.

In studies which examine executive dysfunction in TBI patients, beneficial effects on behavioral outcomes have also been demonstrated with the administration of MPh. Kim et al. (2006) compared TBI patients on MPh and placebo and found that for both visuospatial and WM tasks there were significant improvements in response accuracy for the MPh group. They also found an improvement in RTs for this group but only in the WM task. More recently, improvements in WM, episodic memory and attention were demonstrated in a TBI patient population in a study which combined MPh with a structured metacognitive rehabilitation programme (McDonald et al., 2016).

Neuroimaging studies on WM network changes after the administration of MPh in TBI patients are sparse. Newsome et al. (2009) demonstrated that in an N-back task, the patients in the MPh condition had decreases in activation in several areas of the WM network compared to the placebo condition. These areas included the thalamus, anterior cingulate, cuneus and cerebellum. The suggestion from the modulation in activation is that even a single dose of MPh suppresses activation in areas of the engaged cognitive networks which helps to reduce interference thereby increasing processing efficiency and cognitive control during the task.

In the present study, we aim to further investigate the effect of MPh on a damaged WM network in a TBI patient group by investigating how this cognitive enhancer modulates not only activation levels but also possible alterations in functional connectivity between and within regions of the WM network. We hypothesize that the single dose of MPh will reduce activation levels (as seen in previous studies, Newsome et al., 2009) and that reductions in activation levels will be correlated with improvements in behavioral outcomes. Additionally, we hypothesize that there will be significant changes in the functional connectivity (FC) patterns after the single dose of MPh so that they will more closely resemble those of the HCs.

## Materials and methods

### Setting

We acquired fMRI data while the participants performed an N-back task as part of a double blinded, randomized, crossover, placebo-controlled designed study at the Wolfson Brain Imaging Centre (Cambridge, U.K.).

### Participants

Volunteers with a history of moderate to severe traumatic brain injury (inclusion criteria: age 18–60 years and not recruited to more than three research studies within the calendar year) were referred from the Addenbrooke's Neurosciences Critical Care Unit Follow-Up Clinic, Addenbrooke's Traumatic Brain Injury Clinic and The Royal London Hospital Intensive Care Unit. Patient injuries at admission are described in Table 1. Our sample contains mostly patients with diffuse axonal injuries and small lesions. The patients were sent a written invitation to take part in the study.

**Table 1:** **Injury severity and lesion description for TBI patients**.

**Patient study number**	GCS^*^ on scene/pre-intubation	**Location of injury**	**Time since injury to study scan 1st visit**
2004	5 severe	Scattered petechial hemorrhages in both cerebral hemispheres. A slightly larger hemorrhage in the L temporal lobe (superior to the petrous ridge)	37 months
2001	7 severe	Evidence of hemorrhage in both frontal lobes at gray/white matter interfaces and corpus callosum as well as the superior cerebellar cistern. No mass lesion	25 months
2019	14 mild	R SAH and SDH. Hemorrhagic contusion R posterior temporal lobes. Multiple areas contusion superior frontal lobes and R cerebellar hemisphere, R temporal and inferior frontal lobes	33 months
2011	8 severe	Hemorrhagic contusion L lentiform nucleus. Small focal lesion pons. Bilateral subcortical area frontal lobes. Signal change in corpus callosum	27 months
2002	12 moderate	SAH in the sulci of the L frontoparietal convexity	18 months
2003	5 severe	Multiple hemorrhagic contusions L temporal lobe. Haemorrhage L basal ganglia. R thalamus. R subcortical diffuse axonal injury	14 months
2006	7 severe	Subarachnoid hemorrhage in the L interpeduncular fossa and foramen magnum	32 months
2007	14 mild	Hemorrhagic contusions in orbital frontal cortex. Subarachnoid blood in both cerebral convexities	10 months
2008	3 severe	SAH in convexity sulci bilaterally and interpeduncular fossa	10 months
2010	8 severe	R frontoparietal EDH. Small hemorrhagic contusions L inferior frontal, lateral orbitofrontal gyri and anterior aspect of L temporal lobe	8 months
2012	6 severe	R temporal EDH, hemorrhagic contusions anterior aspect L temporal lobe, posterior inferior R frontal lobe. Scattered areas traumatic SAH in interpeduncular fossa and some of the posterior convexity sulci of both hemispheres	11 months
2013	7 severe	Intraventricular hemorrhage	26 months
2015	Not available—injury abroad	R temporal/parietal contusion	41 months
2016	10 moderate	R SAH and SDH	6 months
2018	3 severe	L temporal lobe contusion and L tentorial SDH	8 months

Thirty-eight volunteers were recruited to the study; 17 (12 male, 5 female) into the TBI arm of the study and 21 (13 male, 8 female) into the healthy control (HC) arm of the study. Two patients were excluded from the analysis (one patient only attended one of the study sessions and the other had excessive movement artifacts in their fMRI scan). We age-matched the HC group to the TBI patient group and for this reason we prospectively excluded six HCs from the data analyses reported here. The patients were at least 6 months post TBI. Four sustained moderate TBI with a score of between 9 and 12 on the Glasgow Coma Scale (GCS) and 11 sustained severe TBI with a GCS score of 8 or below on presentation (see Table 1).

Exclusion criteria included National Adult Reading Test (NART) <70, Mini Mental State Examination (MMSE) <23, left-handedness, history of drug/alcohol abuse, history of psychiatric or neurological disorders, contraindications for MRI scanning, medication that may affect cognitive performance or prescribed for depression, and any physical handicap that could prevent the completion of testing. The mean age of the patient group was 36 years (±13 years). The HC group (mean age of 34 years, ±11 years) were recruited via advertisements in the Cambridge area and were paid for their participation. Cambridgeshire 2 Research Ethics Committee approved the study (LREC 08/H0308/246) and all volunteers gave written informed consent before participating in the study.

### Experimental study design

The study consisted of two visits (separated by 2–4 weeks) for both groups of participants. The TBI volunteers were randomly allocated in a Latin square design to receive one of the two interventions on the first visit (a placebo tablet or 30 mg tablet of MPh), and the alternate intervention on the second visit.

In addition, during the volunteer's first visit, prior to receiving any medication, a series of background assessments (of ~30 min duration) were carried out in both TBI patients and HCs. A baseline cognitive assessment was conducted using a selection of tests from the Cambridge Neuropsychological Test Automated Battery (CANTAB) system (© Cambridge Cognition). These tests were used to establish a baseline cognitive function in both experimental groups and for comparison with the fMRI cognitive tasks if necessary. The tasks selected included tests of WM capacity [Spatial Span (SSP)], episodic memory [Paired Associates Learning (PAL)], executive function (Intra/Extradimensional Set Shift (IED) and Simple Reaction Time (SRT)].

### Methylphenidate

The decision to use 30 mg of MPh was based on comparable doses used in previous studies in healthy participants (Gilbert et al., 2006; Marquand et al., 2011; Costa et al., 2013) as well as NICE guidelines for medication in adults (www.nice.org.uk) which stipulate that when MPh is titrated for side effects and responsiveness in each individual subject, the dose should range from a minimum of 15 mg to a maximum dose of 100 mg. As we were not calculating the dose of MPh by the participant's body weight, we felt it was best to choose an interventional dose at the lower end of the dose range which had also been used in previous studies (Gilbert et al., 2006; Marquand et al., 2011; Costa et al., 2013; Fallon et al., 2017).

After a delay of 75 min to ensure that peak plasma levels of MPh were reached, the volunteers completed a MRI scan which included both fMRI and structural image acquisition. The HC volunteers attended their two fMRI assessments at the same time interval as the patients, but without any pharmacological intervention. HC data reported here is taken from the first study visit with the exception of one statistical comparison (reported in the Behavioral Measures results section) which utilizes HC data from both the first and second visits. This comparison was made in order to confirm that improvement in behavioral outcomes were not due to learning effects.

### N-back WM fMRI task

The N-back (Gevins and Cutillo, 1993) was presented in a block design, where the participants attended the stimuli (single white upper case letters on a black background) on a back-projected visual display. We used a pseudo-randomized block design with 3 blocks of each of the four trial types (0-, 1-, 2-, and 3-back) each of which lasted 36 s and three blocks of fixation (10 s each). During fixation blocks the participants were instructed to fixate their eyes on a white cross in the center of the display screen. For the experimental blocks, letters were presented for 0.5 s each followed by 2.5 s of interstimulus delay. The participants were instructed to respond by pressing the button under their right index finger for targets and the button under their right middle finger for all other non-target letters. Volunteers were required to respond to every stimulus presented as either a target or non-target. Non-responses to the stimuli were not included in the analysis of the behavioral data. The 0-back was treated as a control condition and participants were instructed to respond with their index finger to the target letter “Z” and, as with the other conditions, with their middle finger to non-target letters. Volunteers were introduced to the task procedures and given a practice session on a laptop prior to the fMRI scanning.

The expanded study that was undertaken included tasks that investigated other cognitive domains, including sustained attention, response inhibition and planning. Each participant performed the N-back task in the same fMRI session with three other cognitive tasks (Rapid Visual Information Processing, Stop Signals and Tower of London) which were presented in a randomized order along with a motor task (finger-thumb opposition) which was always first in the task order for each fMRI session. Randomization of the task order avoided the potential confounding effect of fatigue on any single task. As a group, we have recently published our response inhibition results (Moreno-López et al., 2017). We are currently analysing the data from each of the remaining fMRI tasks as this will allow each cognitive domain and its neural correlates to be the focus of the specific analysis.

### Behavioral measures and analyses

Behavioral data analyses were performed using SPSS 21.0 for Windows (SPSS Inc. IL, USA). We used parametric and non-parametric means tests to assess group demographic differences and analyses of variance to assess group performance differences. Using a signal detection framework to differentiate signal from noise, performance accuracy for the N-back task was calculated by determining D′ (Green and Swets, 1966; Swets and Pickett, 1982; Macmillan and Creelman, 2005). This measure was used because it takes into account both the participant's correct response to a target (HITS) and their (incorrect) responses to non-targets (FALSE ALARMS). D′ is a measure of response sensitivity and the participant's ability to correctly detect the target stimulus. Higher positive scores for D′ are an indication of better performance on the task (more HITS, fewer FALSE ALARMS).

Due to ceiling effects for the HCs in the lower cognitive loads (0- and 1-back) and reliability issues due to error variance in the highest cognitive load (3-back) (Hockey and Geffen, 2004; Jaeggi et al., 2010) we report results for the 2-back condition only from both behavioral and activation/connectivity analyses.

### MRI image acquisition

MRI data were acquired on a Siemens Trio 3-Tesla MR system (Siemens AG, Munich, Germany). MRI scanning started with the acquisition of a localizer scan and was followed by a 3D high resolution MPRAGE image [Relaxation Time (TR) 2,300 ms, Echo Time (TE) 2.98 ms, Flip Angle 9°, FOV 256 × 256 mm^2^]. Diffusion Tensor Imaging (DTI) data (63 non-collinear directions, *b* = 1,000 s/mm^2^ with one volume acquired without diffusion weighting (*b* = 0), echo time 106 ms, repetition time 1,700 ms, field of view 192 × 192 mm, 2 mm^3^ isotropic voxels) were also collected for all controls and a subset of patients (*n* = 13) to investigate white matter integrity. Functional imaging data were acquired using an echoplanar imaging (EPI) sequence with parameters TR 2,000 ms, TE 30 ms, Flip Angle 78°, FOV 192 × 192 mm^2^, in-plane resolution 3.0 × 3.0 mm, 32 slices 3.0 mm thick with a gap of 0.75 mm between slices.

### MRI image preprocessing

The DTI data were eddy current corrected and realigned using FSL (fsl.fmrib.ox.ac.uk/fsl). Fractional anisotropy (FA) images were calculated and spatially normalized by utilizing a study specific template constructed in a manner described by our group previously (Stamatakis et al., 2011). The spatially normalized FA images were smoothed with an 8 mm^3^ isotropic Gaussian filter.

The fMRI data were preprocessed and analyzed using Statistical Parametric Mapping (SPM8) software, (Welcome Department of Cognitive Neurology, http://www.fil.ion.ucl.ac.uk/spm/) implemented in MatLab (Mathworks, Sherborn, MA). Preprocessing started with the removal of the first five volumes for each subject to control for initial signal instability. Slice-timing correction was followed by within-subject realignment to correct for movement artifacts. Spatial normalization to the Montreal Neurological Institute (MNI) reference brain was followed by smoothing with an isotropic 6 mm^3^ full-width half-maximal Gaussian kernel.

### DTI statistical modeling

Voxel-wise group FA comparisons between healthy controls and TBI patients were carried out using a two-sample *t*-test. Clusters are reported as significant if they survived family-wise error (FWE) correction for multiple comparisons set at *p* < 0.05 (individual voxel threshold was set at *p* < 0.001 uncorrected). Significant alterations in FA were further examined with MRIcroN software (http://www.mccauslandcenter.sc.edu/mricro/mricron) utilizing the JHU-white matter atlas to annotate significant clusters.

### fMRI statistical modeling

The preprocessed images were entered into a voxel-based model for each volunteer using the general linear model (GLM) framework. Each model contained a regressor with onset times for each stimulus type (0-back, 1-back, etc.) which were convolved with a canonical haemodynamic response function. Six movement parameters obtained at the realignment stage were used as confounds. Low-frequency scanner noise was removed by applying a high-pass filter with a period of 128 s. Contrasts of interest were calculated and the parameter estimate images were entered into a second level group analysis utilizing *t*-tests. The two experimental groups did not differ significantly in terms of age. However, given the documented variability in cerebral perfusion and vascular reactivity between younger and older groups, we included age as a confounding covariate in all group analyses to ascertain that our findings are not caused by variation in age. Results were considered significant if they survived a threshold of *p* < 0.001 (uncorrected) at the voxel level and *p* ≤ 0.05, FWE (corrected for multiple comparisons) at the cluster level. To label significant clusters we used MRIcroN (http://www.mccauslandcenter.sc.edu/mricro/mricron) with the integrated anatomical labeling (Tzourio-Mazoyer et al., 2002) and Brodmann Area templates.

### Relationship between performance and activity in fMRI

We adopted a correlational approach to establish any relationship between changes in activity and changes in performance when comparing the Placebo and Drug conditions in the TBI group. To avoid circularity (Kriegeskorte et al., 2009) we defined Regions of Interest (ROIs) in the HC group around the statistically significant peaks from the 2-back vs. 0-back contrast. For each supra-threshold voxel, a spherical ROI of diameter 6 mm was constructed and mean parameter estimate values from each sphere (Delta parameter estimates) were related to changes in performance (Delta D′).

### Whole brain functional connectivity analysis

The correlational approach above informed a whole brain voxel-wise Psychophysiological Interaction (PPI) analysis, carried out to investigate functional connectivity changes with MPh. The PPI framework allows the extraction of task specific time series and is used to examine how activity in one brain area relates to activity in another brain area in the context of a specific task (Friston et al., 1997). The first level GLM included a task specific time-series for the area of interest as well as 6 movement parameters as confounds. Results are reported at the same threshold levels as specified for the group activation analysis.

## Results

### Behavioral and background measures

There was no significant difference in age between the patient and healthy control groups [*t*_(28)_ = 0.151, *p* = 0.0881]. The TBI patients scored significantly lower than healthy controls on the National Adult Reading Test [*t*_(28)_ = −2.126, *p* = 0.042], which is used as a measure of pre-morbid intelligence (Bright et al., 2002; McGurn et al., 2004). The TBI patients had shorter spatial spans (*U* = 50.5, *p* = 0.009) and longer mean correct latencies in the Simple Reaction Time task [*t*_(28)_ = 3.040, *p* = 0.005] compared to the HCs. The remaining background measures showed no significant differences between the two groups (see Table 2).

**Table 2:** **Differences baseline neuropsychological measures between HCs and TBI patients**.

**SSP**	**PAL total errors**	**IED/EDS errors**	**IED total errors**	**SRT mean correct latency**
*U* = 50.5	*U* = 84	*U* = 104	*U* = 108	*t*_(28)_ = 3.040
*p* = 0.009^*^	*p* = 0.250	*p* = 0.744	*p* = 0.870	*p* = 0.005^**^

* *Significant at p < 0.05*.

** *Significant at p < 0.005*.

*SSP, Spatial Span length; PAL, Paired Associates Learning; IED/EDS, Intra/Extra Dimensional Shift; IED, Intra Extra Dimension; SRT, Simple Reaction Time. SSP, PAL, IED, SRT tasks administered as part of the baseline neuropsychological assessment on the CANTAB system*.

For the 2-back level of difficulty, one-way MANOVAs were performed to assess performance differences in D′ between experimental groups. Univariate between group tests revealed a significant difference in D′ [*F*_(1, 28)_ = 4.426, *p* < 0.05 η_*p*_^2^ = 0.136] when HCs were compared to the TBI patients on Placebo. A comparison between the HC group and TBI patients on MPh was not significant [*F*_(1, 28)_ = 3.665, *p* = 0.66], suggesting a MPh mediated normalization in performance.

Next, we examined a possible relationship between time since injury/severity of injury and performance. For the TBI group, there was no correlation with the time since injury and D′ performance [*r*_(13)_ = −0.023, *p* = 0.934] but there was a significant positive correlation with D′ and injury severity (measured by initial GCS score) [*r*_(12)_ = 0.582, *p* = 0.029].

Although we implemented a randomization procedure in the administration of placebo/drug in the patients, we further ascertained that improvement in performance could not be explained by learning effects by comparing D′ in the HC group between the two study visits (HCs visited twice without pharmacological intervention). A Wilcoxon signed rank test showed that there was not a significant difference in performance between the first and second study visit for the HCs (*Z* = −0.745, *p* = 0.456).

**Figure 1:**
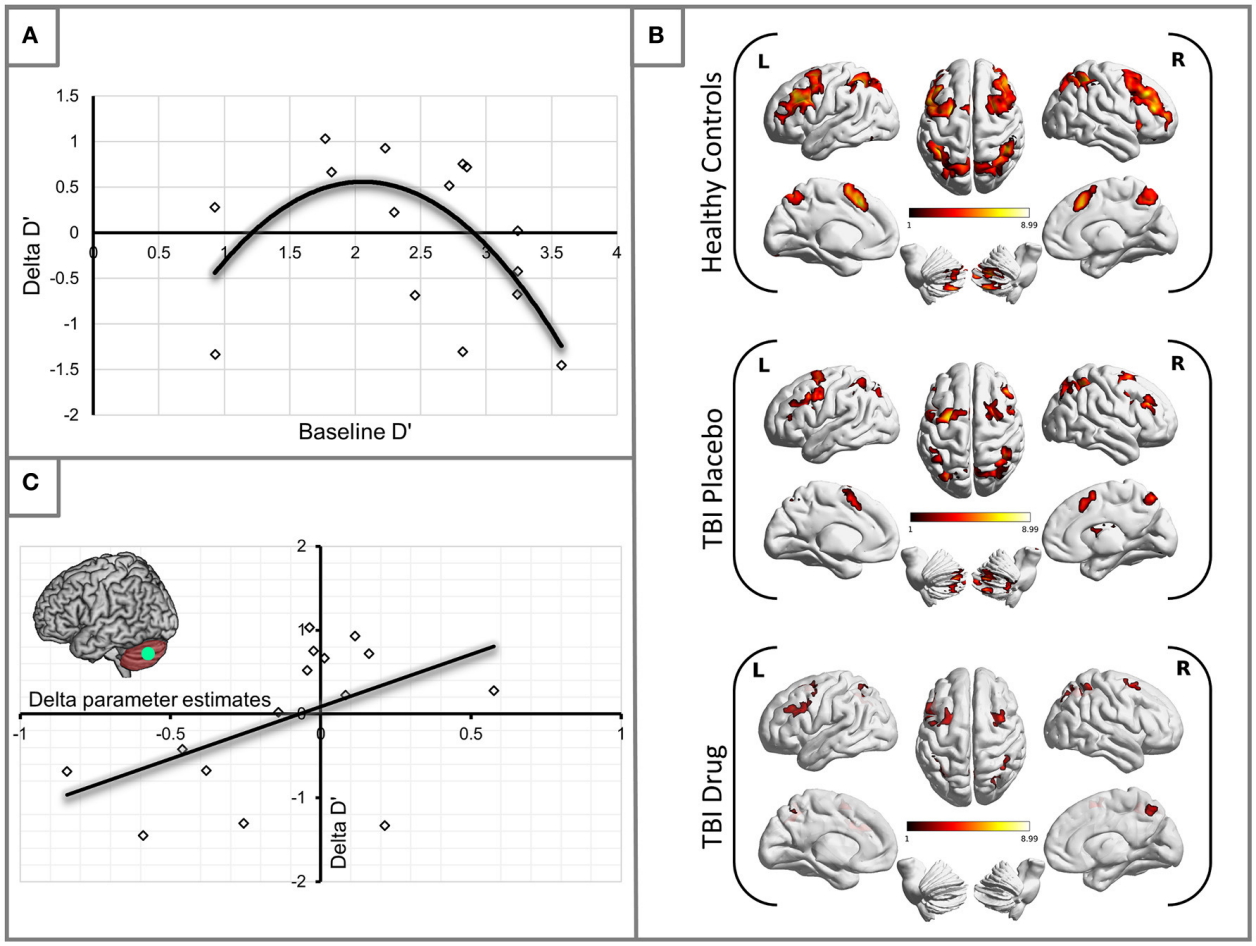
**(A)** Methylphenidate effect on the TBI group performance. The plot demonstrates changes in D′ for the 2-back load. Delta D′ represents the change between D′ Drug and D′ Placebo and baseline D′ is the D′ measured during the placebo arm of the study. We observed the greatest behavioral benefit in patients with baseline performance within the middle range of D′ scores (*R*^2^ = 0.387). **(B)** Significant activity for the 2-back vs. 0-back contrast by experimental group (see Table 3 for statistical peaks). Results are superimposed on a template supplied by MRIcroGL (http://www.mccauslandcenter.sc.edu/mricrogl/). **(C)** Significant positive correlation of change in activation (x-axis) in the LCb ROI with change in performance by D′ for the TBI patient groups (*R*^2^ = 0.259).

Having established some performance changes in the TBI group following the administration of MPh we set out to ascertain whether there was uniform performance improvement or otherwise in the group. To this end, we plotted changes in D′ (Delta D′ = D′ TBI Drug – D′ TBI Placebo) against the patients' baseline (placebo) D′. A second-order polynomial curve in the shape of an inverted U fitted this relationship (see Figure 1A). Our finding demonstrates that patients in the middle range of baseline performance showed the most benefit from MPh.

### Group activation analyses (2-back vs. 0-back)

The HC group demonstrated robust activation in all areas associated with the WM network (Owen, 1997; Fletcher and Henson, 2001) including bilateral frontal, bilateral parietal, cerebellar and premotor areas. Large clusters of activation were found in middle frontal areas of both hemispheres and left hemispheric inferior frontal and mid frontal areas (Supplementary Motor Area). Activation in similar areas was demonstrated for the TBI patients on drug and placebo but the patient activation was not as extensive as for the HCs (Figure 1B, Table 3).

**Table 3:** **Significant activation peaks for the 2-back vs. 0-back contrast for each experimental group**.

**Subject group**	**Cluster level**	**Cluster extent**	**MNI co-ordinates**			***t***	**BA**	**Peak in cluster**
	**p (FDR)**		***x***	***y***	***Z***			
**CONTROLS**								
	0.000	1181	9	23	46	8.58	32	R Superior Medial Frontal
			0	20	46	8.56	32	L Supplementary Motor Area
			−24	5	58	8.49	6	L mid Frontal
			−39	8	28	4.74	44	L Inferior Frontal Operculum
			−48	32	37	4.54	45	L mid frontal
	0.000	726	48	41	19	7.94	45	R mid Frontal
			42	11	52	4.35	9	R mid Frontal
			27	23	61	3.86	8	R Superior Frontal
			48	20	34	3.74	44	R Inferior Frontal Operculum
	0.000	593	−33	−49	46	8.91	40	L Inferior Parietal
			−33	−58	52	7.93	7	L Inferior Parietal
			45	−40	46	7.89	40	R Supramarginal Gyrus
			42	−46	52	7.67	40	R Inferior Parietal
			30	−64	52	6.30	7	R Superior Parietal
	0.000	282	−6	−64	58	5.35	7	L Precuneus
			9	−67	49	5.30	7	R Precuneus
	0.000	222	30	−73	−50	8.99	n/a	R Cerebellum 7b
			36	−61	−32	7.48	n/a	R Cerebellum Crus 1
	0.000	117	−30	−70	−50	8.75	n/a	L Cerebellum 7b
			−33	−61	−32	5.80	n/a	L Cerebellum Crus 1
	0.005	75	36	23	10	6.11	48	R inferior frontal Triangularis
			39	23	−2	5.43	47	R Insula
**PLACEBO**								
	0.000	343	42	−46	52	7.44	40	R Inferior Parietal
			39	−73	37	5.33	19	R mid Occipital
			33	−64	49	5.05	7	R Angular Gyrus
	0.000	270	−27	5	55	8.82	8	L mid Frontal
			−24	−4	55	7.33	6	L Superior Frontal
			−6	11	52	7.08	6	L Supplementary Motor Area
			6	20	52	5.20	6	R Supplementary Motor Area
			3	20	43	4.55	32	R mid Cingulum
	0.000	168	−48	−1	40	11.96	6	L Precentral
			−39	23	31	5.98	44	L Inferior Frontal Triangularis
			−39	32	34	5.69	45	L mid Frontal
	0.000	160	27	8	64	6.65	6	R Superior Frontal
			30	11	52	4.95	8	R mid Frontal
	0.000	109	−36	−70	−32	6.74	n/a	L Cerebellum Crus 1
			−24	−67	−41	4.36	n/a	L Cerebellum 7b
	0.001	89	33	−70	−32	7.91	n/a	R Cerebellum Crus 1
	0.005	66	−30	−73	37	6.22	7	L mid occipital
	0.011	54	51	5	40	5.91	6	R Precentral
			36	2	28	4.85	48	R Inferior Frontal Operculum
	0.013	49	9	−67	55	6.62	18	R Precuneus
**DRUG**								
	0.001	105	−48	−46	40	5.45	40	L Inferior Parietal
			−30	−64	37	3.43	19	L mid Occipital
	0.005	72	42	−46	43	5.02	40	R Inferior Parietal
			48	−37	49	4.75	7	R Inferior Parietal
	0.023	47	24	8	58	4.89	6	R Superior Frontal
			39	8	61	4.84	6	R mid Frontal

### Relationship between performance and activity

Our behavioral analysis demonstrated an improvement in performance for patients in the middle range of baseline performance. To investigate which specific areas of the WM network mediated this change in performance, we carried out a correlational analysis relating performance changes (D′ TBI Drug – D′ TBI Placebo), to activation changes (TBI Drug-TBI Placebo). We found that changes in left cerebellum (LCb) activity significantly positively correlated with changes in the D′ performance (*r* = 0.509, *df* = 13, *p* = 0.05, see Figure 1C).

### Whole brain functional connectivity analysis

We investigated next how the LCb interacts with the rest of the brain during the execution of the WM task and how these interactions maybe altered by MPh.

#### Connectivity from the left cerebellum (LCb) 7b (x−30 y−70 z−50)

**Figure 2:**
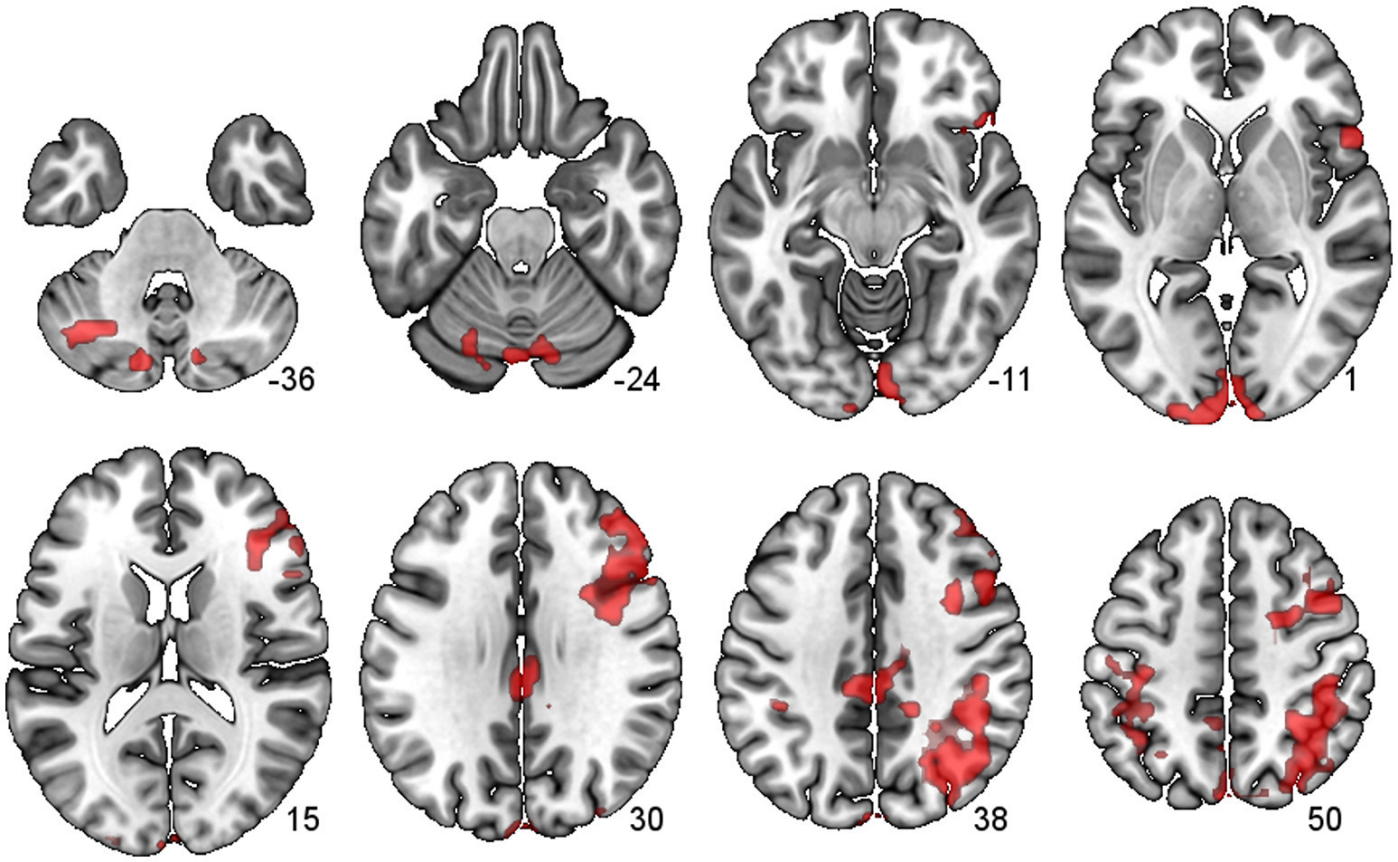
**PPI Connectivity for the TBI Placebo condition from the LCb ROI**. Areas that displayed negative PPI connectivity with the LCb ROI. The data shown are from axial slices at z −36, −24, −11, 1, 15, 30, 38, and 50 mm at MNI space.

There were several large clusters that demonstrated negative connectivity with the LCb in the TBI Placebo condition, signifying reduced connectivity of the condition of interest (2-back) in comparison to baseline. The clusters encompassed contralateral posterior cortical areas extending to the inferior parietal cortex and angular gyrus as well as clusters in motor, inferior frontal and subcortical areas. The HCs and TBI patients on MPh did not display any significant negative connectivity (see Table 4 and Figure 2).

**Table 4:** **Significant connectivity peaks for the Left Cerebellum (LCb) for the 2-back**.

**LCb (x−30, y−70, z−50)**	***P*-value (cluster)**	**Extent**	***x***	***y***	***z***	**t-score**	**BA**	**Peak in cluster**
	0.000	699	45	−43	40	8.14	40	R Inf Parietal
			45	−64	40	6.37	39	R Angular Gyrus
			42	−31	46	5.59	2	R Postcentral
			30	−61	40	4.60	19	R mid Occipital
			15	−49	46	4.04	n/a	R Precuneus
		599	42	5	46	5.98	6	R Precentral
			51	32	31	5.76	45	R Inf Frontal Triangularis
			39	17	31	5.14	44	R Inf Frontal Operculum
			39	23	22	5.12	47	R Insula
		345	−3	−103	4	7.75	7	L Calcarine
			12	−76	58	5.04	7	R Precuneus
			−6	−76	55	4.99	7	L Precuneus
			0	−88	37	4.67	19	L Cuneus
			6	−94	28	4.65	19	R Cuneus
		260	−33	−19	61	5.55	6	L Precentral
			−33	−55	64	3.96	7	L Superior Parietal
			−48	−22	49	3.41	3	L Postcentral
			−45	−49	49	3.06	40	L Inferior Parietal
	0.011	157	3	−28	34	5.33	23	R mid Cingulum
			−6	−31	40	4.34	23	L mid Cingulum
	0.049	115	27	8	−17	5.13	34	R Amygdala
			48	23	−8	5.05	38	R Inf Frontal Orbitalis
			54	17	1	4.75	48	R Inf Frontal Operculum
			39	17	−5	4.19	47	R Insula

### Structural connectivity findings

**Figure 3:**
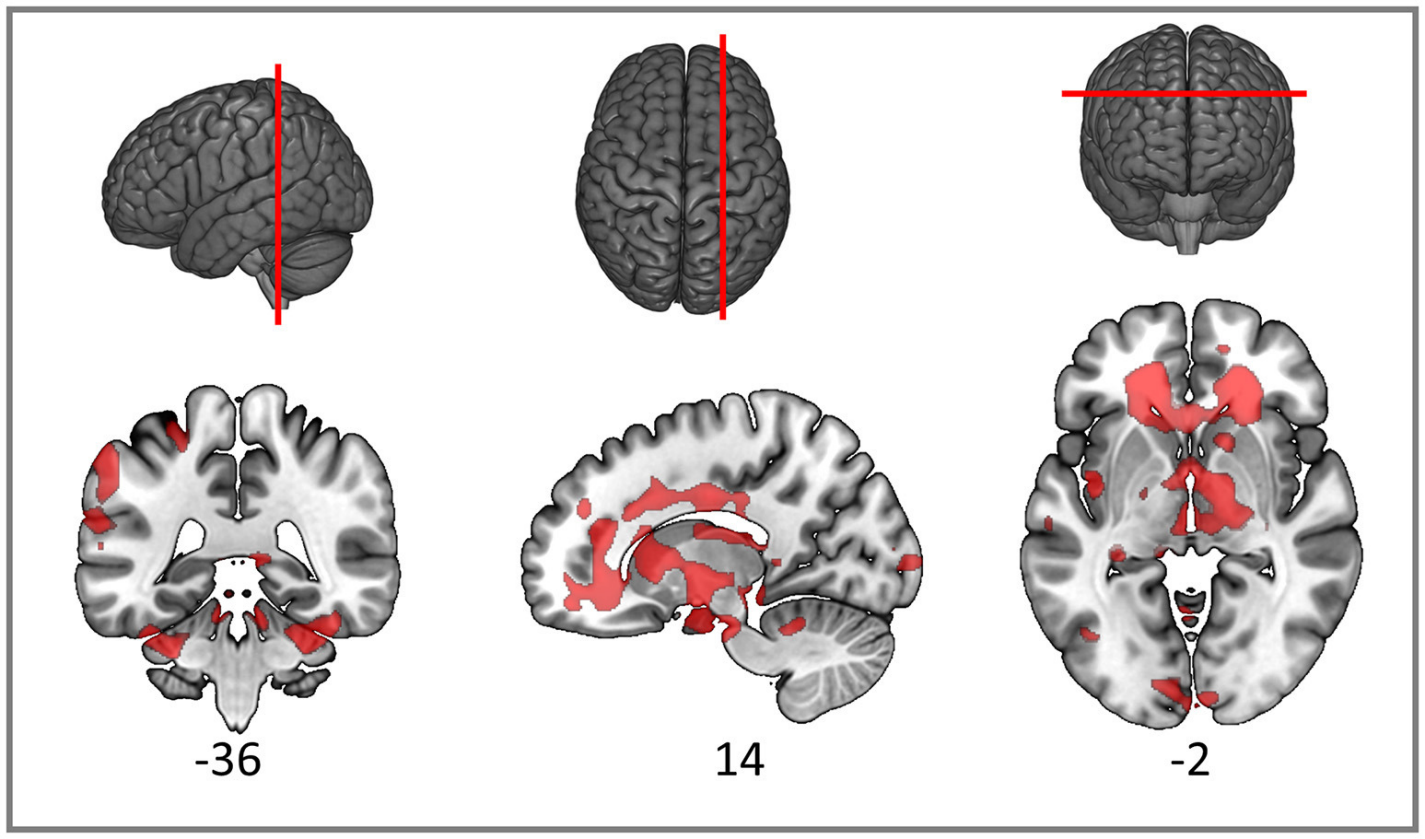
**Red clusters on the orthogonal T1 weighted slices demonstrate widespread FA reductions in TBI patients compared to healthy controls**. For cluster peak descriptions see Table 5.

As expected, the TBI patients demonstrated widespread FA reductions with significant clusters in the inferior parietal cortex, widespread frontal areas (particularly orbitofrontal and postcentral), caudate nucleus, putamen, thalamus and bilateral occipital cortices. Specific white matter tracts implicated were the right internal capsule, the right posterior and anterior arcuate segments, the anterior and posterior corpus callosum, the right corticospinal tract and cerebral and cerebellar peduncles (Figure 3). White matter loss in TBI patients has been previously reported not only in the supratentorial compartment but also the cerebellum and brainstem (Newcombe et al., 2016). The structural findings provide some foundation for the functional connectivity changes we reported in the previous section.

**Table 5:** **Significant peaks for the comparison of FA healthy controls > patients**.

**Cluster level**	**Cluster extent**	**MNI co-ordinates**			***t***	**Peak in cluster**
**p (FDR)**		***x***	***y***	***Z***		
0.000	23,760	−55	−26	41	8.37	L supramarginal G
		−56	−6	12	7.72	L rolandic Oper
		−62	−21	22	5.66	L middle temporal
0.000	81,165	13	−6	−5	7.58	R cerebral peduncle
		−26	−35	−33	7.03	L Middle cerebellar peduncle
		−3	−12	−15	6.72	L cerebral peduncle
0.050	2,719	−41	−3	−2	6.97	L external capsule
		−33	4	−20	4.8	L superior temporal pole
		−39	10	−24	4.4	L superior temporal pole
0.001	6,078	−23	−78	32	5.97	L superior occipital
		−30	−90	14	5.19	L middle occipital
		−23	−91	27	5.16	L superior occipital
0.001	6,139	−16	35	34	5.74	L anterior coronal radiata
		−22	43	20	5.48	L anterior coronal radiata
		−15	25	38	5.36	L superior coronal radiata
0.035	3,076	3	−96	0	4.67	R calcarine
		17	−98	5	4.58	R calcarine
		−8	−91	−3	4.58	L calcarine

## Discussion

Our behavioral findings compare favorably with previous studies demonstrating performance deficits in TBI patients (Perlstein et al., 2004; Kasahara et al., 2011). Importantly, our proof of concept study demonstrated an improvement in performance following the administration of MPh. Our study supports the involvement of the cerebellum in functional normalization since we found that, following the administration of MPh, performance improvement in patients was positively correlated with changes in the activation levels in the LCb. Patients on placebo showed both structural and functional connectivity changes between LCb and inferior parietal cortex as well as inferior frontal and subcortical areas considered important for the execution of the WM task (Smith et al., 1996; Ravizza et al., 2004). Critically, some of these connectivity changes appear to be restored when patients took a single dose of MPh. These network level integration changes in the TBI WM network provide some additional evidence of the important role the cerebellum plays in higher cognitive function and WM in particular (see Buckner, 2013 for review).

### The role of the cerebellum in WM

The cerebellum has been recently recognized to contribute extensively to cognition, emotion and affect in humans. The posterior lobe of the cerebellum processes cognitive and emotional information (Stoodley and Schmahmann, 2009; Stoodley, 2012) and can be segregated anatomically by cognitive function; the afferents from the cerebral cortex to the cerebellum are also targets of cerebellar output, creating closed loops for cognition. Allen et al. (2005) conducted a whole-brain functional connectivity analysis using seed regions in the bilateral cerebellar dentate nucleus in healthy volunteers. They found that the left cerebellar dentate was functionally connected to the right inferior parietal lobe, the right superior frontal gyrus and bilateral middle frontal gyri. Considering this evidence in the context of our findings we see that the posterior areas of the cerebellum are functionally connected to regions which are integral to the WM network and are the same areas in which we find compromised connectivity in patients on Placebo.

One of the roles attributed to the cerebellum is that it functions as a “comparator” performing error adjustments in executive processing that may be key to behavioral deficits seen in our TBI patients on placebo (Koch et al., 2007; Leung et al., 2007; Oliveri et al., 2007). The cerebellum is involved in attention, timing monitoring/adjustment (Nichelli et al., 1996; Mangels et al., 1998; Ivry and Spencer, 2004; Hayter et al., 2007) and serial ordering of information (Henson et al., 2000). These functions of the posterior cerebellum are postulated to act together as an error-prediction system (Dreher and Grafman, 2002; Ben-Yehudah et al., 2007) which starts with the assimilation of incoming sensory information from the visual cortex then feeding forward to the thalamus and on to the frontal and parietal regions for higher level cognitive processing (Middleton and Strick, 1997; Schmahmann and Pandya, 1997). Disruption of any of the steps in this cognitive pathway would effectively breakdown the feed-forward and feedback loops between the cerebellum and cerebral cortex. Any such disruption would result in the inability to effectively keep track of timing or order of the incoming stimulus. Once this temporal order is compromised, it would be difficult to co-ordinate the acquisition and updating of the new information because there is no longer a point of reference for error adjustment purposes. For the N-back task, this disruption would result in deficits in the ability to monitor and update the letters being presented sequentially, thus weakening the quality of information reaching the frontal lobes resulting in a detrimental effect in performance.

### Proposed Mph action on cerebellar/striatal interaction

**Figure 4:**
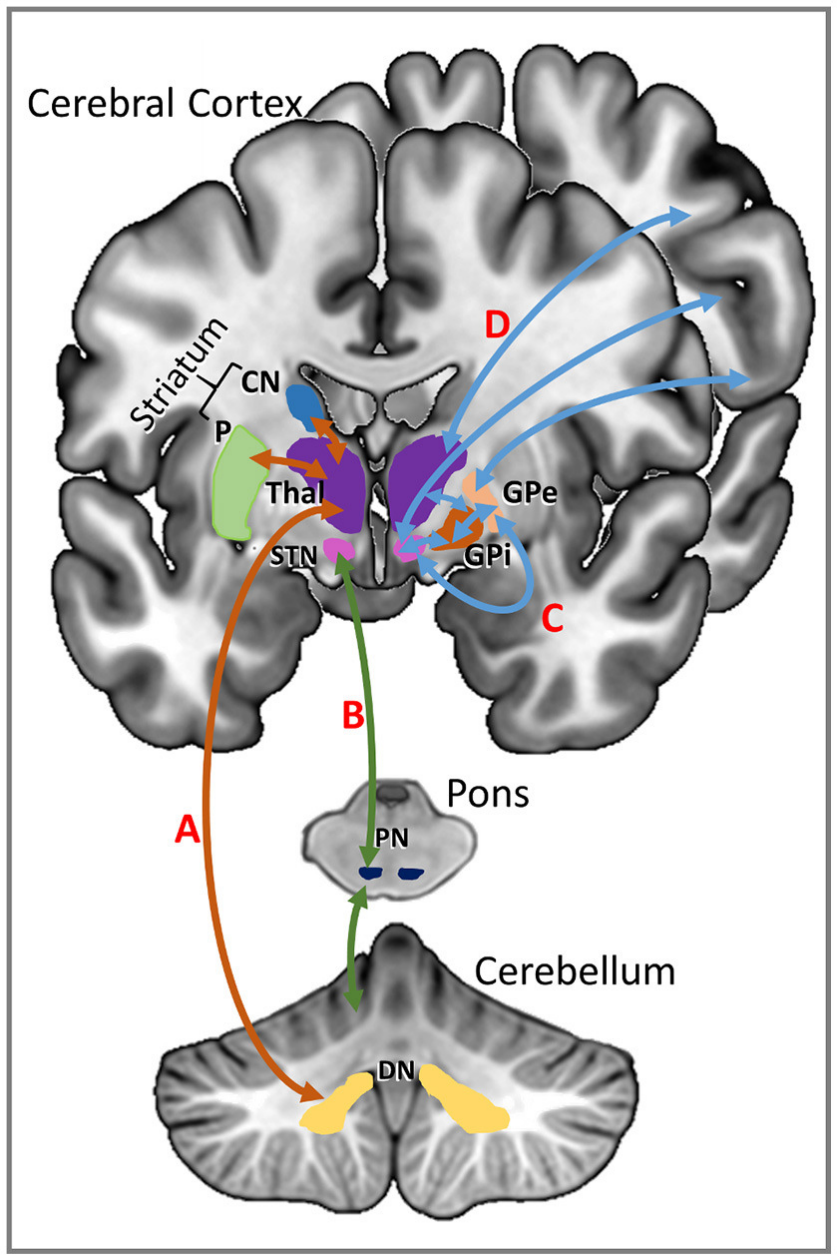
**Communication loops between the striatum and the cerebellum shown on the left of this schematic representation. (A)** Striatal input from the caudate nucleus (CN) and putamen (P) (shown in orange) is routed via the thalamus (Thal) to the dentate nucleus (DN) of the cerebellum. **(B)** Input from the subthalamic nucleus (STN) of the basal ganglia is directed through the pontine nuclei (PN) of the pons to the cerebellar cortex (shown in green). Communication pathways between the basal ganglia and the cerebral cortex are shown in blue on the right of the schematic. **(C)** The subthalamic nucleus has direct connections to the cerebral cortex as well as indirect connections to the cerebral cortex via thalamus and the globus pallidus (GP). **(D)** The thalamus has direct reciprocal connections with the cerebral cortex.

MPh acts as a dopamine reuptake inhibitor by blocking dopamine transporters in the striatum, which increases extracellular levels of dopamine in the striatum and the frontal cortex (Volkow et al., 2001; Arnsten and Li, 2005; Aarts et al., 2011). Both the basal ganglia and cerebellum have separate, parallel connections with the cerebral cortex but they also communicate with each other, mainly through the subthalamic nucleus (STN) (Parent and Hazrati, 1995b; Brown et al., 2001; Williams et al., 2002; Aravamuthan et al., 2007) and the dentate nucleus of the cerebellum (Middleton and Strick, 1994; Hoshi et al., 2005). There is a reciprocal connection from the STN to the input stages of the cerebellum via the pontine nuclei which creates a communication loop between the two structures (D'Angelo and Casali, 2013). The other reciprocal connection is from the dentate nucleus and the striatum via the thalamus; the thalamus is also the gateway to the cerebral cortex from the basal ganglia (Parent and Hazrati, 1995a; Allen et al., 2005; Haber and Calzavara, 2009). The increased levels of excitatory dopamine in the striatum resulting from the administration of MPh may be acting to increase the level of activity in these communication loops (see Figure 4 for an illustration).

When considering the changes in connectivity we observed between the cerebellum and the frontal and parietal cortices after a dose of MPh, it becomes clear that the connections to the basal ganglia and the cerebellum may be influencing the levels of integration within the entire WM network. The error adjustment and monitoring role of the cerebellum is executed via the anatomical and functional connections (Middleton and Strick, 2000b; Allen et al., 2005; D'Angelo and Casali, 2013) to the prefrontal and parietal cortices (particularly via the dentate nucleus) but there are also the indirect pathways to the cerebral cortex from the dentate nucleus through the striatum, particularly the caudate nucleus and the putamen. There is evidence for the specific involvement of the caudate nucleus in WM capacity (Landau et al., 2009) and manipulation of information in WM (Lewis et al., 2004). The increase in dopamine within this reciprocal cerebellar/basal ganglia loop may modulate the strength of functional connectivity—an inference supported by our functional connectivity analyses.

WM can be thought of as a system based on feedback loops between sensory processing areas in the cerebellum feeding information through to the striatum, the thalamus, cerebral cortex and then, in reverse order, back to the cerebellum. This system of constant monitoring and updating keeps the frontal cortex up-to-date with incoming information to enable accurate decision-making. In a damaged network, the timing and updating mechanism may not be fully functioning in the first steps of the system between the cerebellum and the basal ganglia. Evidence for this error-detection mechanism is provided in a study by Dreher and Grafman (2002) which used a task-switching experiment with variable timing in healthy volunteers. They found that the unpredictability of the task order activated the anterior basal ganglia (the putamen specifically) and the irregularity of the timing for the task activated the cerebellum. The dentate nucleus of the cerebellum has anatomical connections to the putamen and the caudate (Bostan et al., 2010, 2013) so if white matter damage (widespread in DAI patients) disrupts the feedback system in the early stages, the frontal and parietal cortices will not be activated coherently in order to achieve successful WM task performance.

### Individuals benefit differentially from methylphenidate—potential approaches to treating TBI deficits

The relationship that we show between baseline D′ and changes in D′ with methylphenidate in Figure 1A suggests that the effects of methylphenidate on behavioral outcomes are not uniform between subjects. The shape of the relationship between these two variables recapitulates the shape of the dose-response curve found when levels of dopamine in the Prefrontal Cortex (PFC) were related to cognitive performance in earlier animal studies (including WM paradigms) (Goldman-Rakic et al., 2000; Seamans and Yang, 2004; Vijayraghavan et al., 2007) as well as studies of healthy human populations (Cools and Robbins, 2004; Cools and D'Esposito, 2011). The implication from these earlier studies was that extremes in extracellular dopamine in the PFC (either too much or too little) result in impairment in cognitive performance in healthy volunteers; the peak benefit in cognitive performance was achieved when the dopamine levels were optimized for the subject's basal dopamine level (Cools and Robbins, 2004).

This background provides an explanation for the right part of our curve, but does not satisfactorily explain the downstroke on the left, since patients with the worst baseline performance (and lowest D′) might be expected to benefit most from any modulation of dopaminergic tone. It may be that the reduction in benefit at this end of the curve represents patients in whom structural connectivity is so severely compromised that neurochemical enhancement of residual function cannot deliver significant benefit. Notwithstanding the explanation for our findings, these data provide an important insight into stratification of patients for treatment with methylphenidate in this context—suggesting that those patients with moderately severe cognitive deficits may be most likely to benefit from therapy. This hypothesis is testable in subsequent studies.

To date, the majority of studies investigating cognitive and neural effects of MPh have used a single dose of MPh in both patient and healthy populations. Changes in connectivity and activation were demonstrated after a single dose in HCs in both task-based (Tomasi et al., 2011) and resting state fMRI studies (Mueller et al., 2014). Ramasubbu and Goodyear (2008) carried out a study in a cohort of stroke patients which involved periodic fMRI scanning with two cognitive tasks during a 3-day course of MPh administration and they found increases in activation in the patient WM networks. McDonald et al. (2016) found that TBI patients who received a dose of MPh in conjunction with a neurocognitive rehabilitation programme demonstrated improvements in several cognitive domains including WM. Future TBI studies designed to have a daily dose of MPh, periodic cognitive testing and serial MRI scans would add to the current knowledge of the longer term neural activation patterns and pharmacological action of MPh.

Future studies aiming to further clarify the biological mechanisms of methylphenidate on damaged neural networks should also endeavor to recruit a much larger cohort of TBI patients who were recovering from severe head injury (initial GCS <8). The heterogeneity of this particular cohort makes it difficult to generalize type of injury for analysis purposes but with a larger number of recruited patients having sustained a severe head injury, the statistical power would be improved along with identification of the differences in behavioral outcomes within the TBI groups.

Likewise, it would be useful to have had the HC group be randomized to the drug and placebo arms of the study in the same procedure as the TBI patients. Such design would enable the behavioral and connectivity analysis in the uninjured brain, which would provide a more structured comparison of the network changes that are seen in the TBI patient group after a single dose of methylphenidate.

## Conclusion

Our study demonstrates how a compromised WM network in TBI patients partially recovers function by the administration of a neurocognitive enhancer and this is reflected by improvements in behavioral performance, activity profiles as well as connectivity changes. Our key finding is that activity changes in the left cerebellum are related to changes in performance in a group of TBI patients. This functional integration enhancement from the cerebellum to the rest of the WM network after a dose of MPh may be facilitating residual functionality and, consequently, an error-adjustment mechanism which results in performance accuracy improvements for the TBI patients. The action of MPh may be providing a degree of cognitive control in these areas of the TBI WM network. The modulation of the activation and integration of the WM network as influenced by a single dose of MPh may be seen as an approximation of the natural recovery of function over time.

## Ethics statement

This study was carried out in accordance with the recommendations of the Cambridgeshire 2 Research Ethics Committee in accordance with the Governance Arrangements for Research Ethics Committees (July 2001) and complies fully with the Standard Operating Procedures for Research Ethics Committees in the United Kingdom. All subjects gave written informed consent in accordance with the Declaration if Helsinki. The protocol was approved by the Cambridgeshire 2 Research Ethics Committee and the Cambridge University Hospitals NHS Foundation Trust Research and Development Department.

## Author contributions

DM, BS, and ES conceived and designed the study. AM recruited the patients. AM and ES collected the data and undertook the analysis of the data. AM wrote the first draft of the manuscript with contributions from ES. All authors have contributed to the manuscript, helped with a series of revisions and approved the final manuscript for publication.

### Conflict of interest statement

The authors declare that the research was conducted in the absence of any commercial or financial relationships that could be construed as a potential conflict of interest.
